# Electronic Sensors for Assessing Interactions between Healthcare Workers and Patients under Airborne Precautions

**DOI:** 10.1371/journal.pone.0037893

**Published:** 2012-05-25

**Authors:** Jean-Christophe Lucet, Cédric Laouenan, Guillaume Chelius, Nicolas Veziris, Didier Lepelletier, Adrien Friggeri, Dominique Abiteboul, Elisabeth Bouvet, France Mentre, Eric Fleury

**Affiliations:** 1 Infection Control Unit, Bichat-Claude Bernard Hospital, Assistance Publique-Hôpitaux de Paris, Paris, France; 2 Univ Paris Diderot, Sorbonne Paris Cité, Paris, France; 3 Institut National de la Santé et de la Recherche Médicale U738, Paris, France; 4 Service de Biostatistiques, Bichat-Claude Bernard Hospital, Assistance Publique-Hôpitaux de Paris, Paris, France; 5 Laboratoire de l’Informatique du Parallélisme, Unité Mixte de Recherche 5668, Ecole Normale Supérieure, Institut National de Recherche en Informatique Appliquée, Université Claude Bernard 1, Lyon, France; 6 Equipe d’Accueil 1541, Université Pierre et Marie Curie, Paris, France; 7 Laboratoire de Bactériologie-Hygiène et Centre National de Référence des Mycobactéries et de la Résistance des Mycobactéries aux Antituberculeux, Hôpital Pitié-Salpêtrière, Assistance Publique-Hôpitaux de Paris, Paris, France; 8 Department of Occupational Health, Bichat-Claude Bernard Hospital, Assistance Publique-Hôpitaux de Paris, Paris, France; 9 Infectious Diseases Department, Bichat-Claude Bernard Hospital, Assistance Publique-Hôpitaux de Paris, Paris, France; INSERM & Universite Pierre et Marie Curie, France

## Abstract

**Background:**

Direct observation has been widely used to assess interactions between healthcare workers (HCWs) and patients but is time-consuming and feasible only over short periods. We used a Radio Frequency Identification Device (RFID) system to automatically measure HCW-patient interactions.

**Methods:**

We equipped 50 patient rooms with fixed sensors and 111 HCW volunteers with mobile sensors in two clinical wards of two hospitals. For 3 months, we recorded all interactions between HCWs and 54 patients under airborne precautions for suspected (n = 40) or confirmed (n = 14) tuberculosis. Number and duration of HCW entries into patient rooms were collected daily. Concomitantly, we directly observed room entries and interviewed HCWs to evaluate their self-perception of the number and duration of contacts with tuberculosis patients.

**Results:**

After signal reconstruction, 5490 interactions were recorded between 82 HCWs and 54 tuberculosis patients during 404 days of airborne isolation. Median (interquartile range) interaction duration was 2.1 (0.8–4.4) min overall, 2.3 (0.8–5.0) in the mornings, 1.8 (0.8–3.7) in the afternoons, and 2.0 (0.7–4.3) at night (*P*<10^−4^). Number of interactions/day/HCW was 3.0 (1.0–6.0) and total daily duration was 7.6 (2.4–22.5) min. Durations estimated from 28 direct observations and 26 interviews were not significantly different from those recorded by the network.

**Conclusions:**

The RFID was well accepted by HCWs. This original technique holds promise for accurately and continuously measuring interactions between HCWs and patients, as a less resource-consuming substitute for direct observation. The results could be used to model the transmission of significant pathogens. HCW perceptions of interactions with patients accurately reflected reality.

## Introduction

Most of the published data on interactions between patients and healthcare workers (HCWs) in the hospital were obtained by direct observation of samples of patients and/or HCWs. Direct observation is tedious, time-consuming, and costly and is therefore feasible only over short periods. Therefore, the results may fail to accurately reflect interactions between patients and HCWs.

Despite progress in prevention and treatment, tuberculosis (TB) remains a major public health problem in the developing world. In industrialized countries, the incidence of TB is declining and public health authorities now focus on specific interventions such as early diagnosis and treatment or contact detection when a TB case is identified. Improvements in TB infection control in hospitals over the last two decades [1] have decreased the incidence of HCW contamination due to contact with TB patients [2].

However, there is a need for additional information about occupational TB, in order to improve infection-control measures. For example, data on risk factors for acquiring latent TB from an index patient are limited, as they derive only from outbreak investigations and expert opinion [1]. Similarly, little is known about HCW exposure to patients with active TB. Assessment of the latent TB risk in HCWs relies on several factors, including the presence in the index patient of a cough and/or of lung involvement with acid-fast bacilli (AFB) in sputum smears, estimated infectiousness of the patient, use of aerosol-generating procedures, and exposure time without personal protective equipment [1]. Exposure time is evaluated based on recollection by the HCW of the number and duration of contacts with the patient, which may be inaccurate. Likewise, the exposure time cutoff above which an investigation is initiated varies across countries [3].

To address these limitations and to obtain accurate data on interactions between patients and HCWs, radiofrequency identification (RFID) systems are being developed. We used a RFID to continuously and accurately measure interactions between inpatients with suspected or confirmed TB and HCWs exposed to these patients over a 3-month period. Our objectives were (i) to assess the pattern of interactions between TB patients placed under airborne precautions and HCWs, and (ii) to compare the accuracy of interaction data obtained using three surveillance systems, i.e., the RFID, direct observation, and interviews about HCW perceptions.

**Figure 1:**
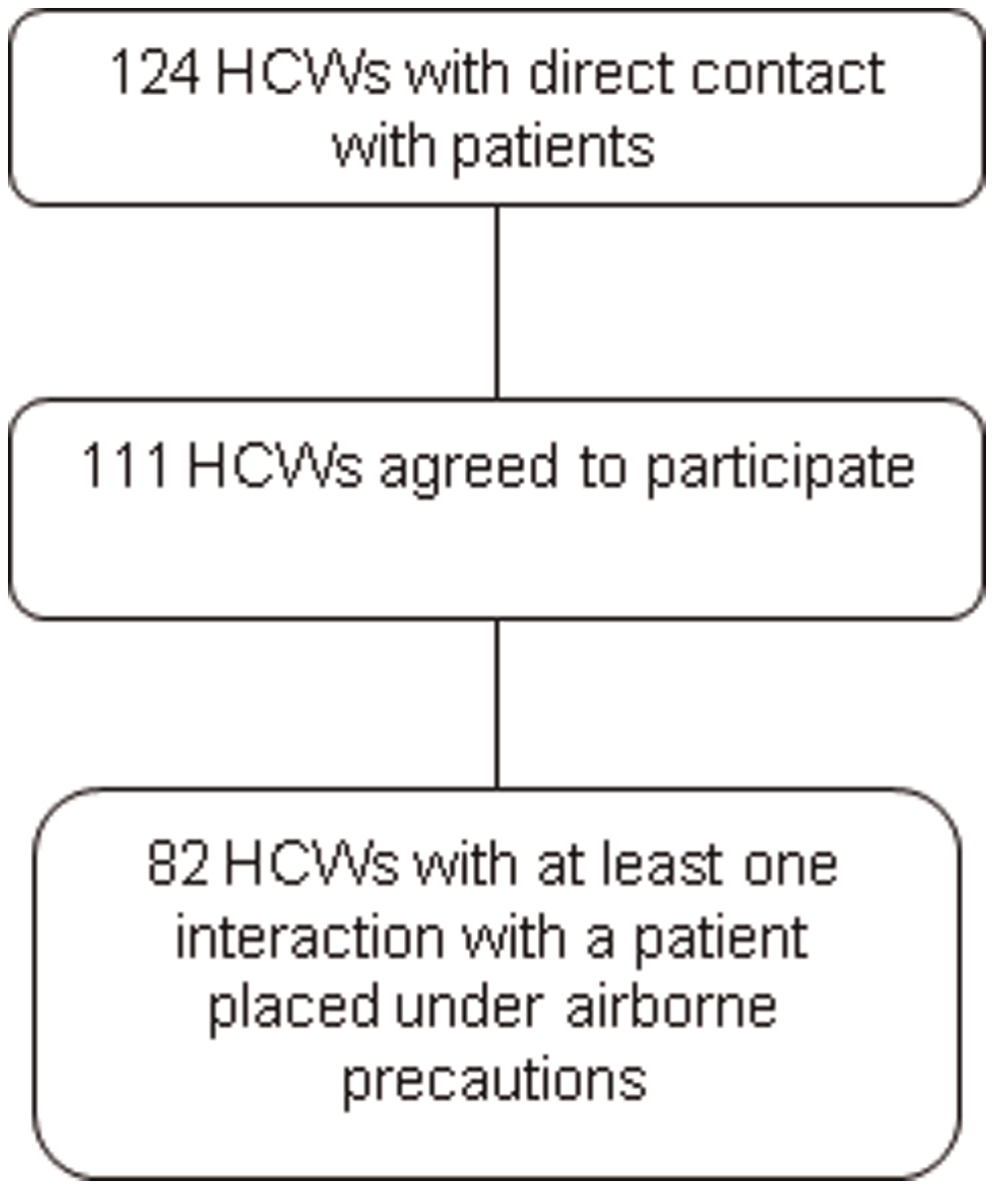
Flow-chart of healthcare workers participating in the study.

## Methods

### Study Design

This was an observational, prospective, study in two centers. HCWs were recruited from two clinical wards that regularly admitted patients with TB. One clinical unit was a 34-bed, 32-room ward in the infectious diseases department at the Bichat - Claude Bernard Teaching Hospital, Paris, France. The other unit was a 25-bed, 18-room ward in the pulmonology unit of the Pitié-Salpêtrière Teaching Hospital, Paris, France. In both wards, all HCWs who had regular direct contact with TB patients were invited to participate in the study, on a volunteer basis. Volunteering HCWs signed a consent form.

The study was approved by the ethics committee of the Bichat-Claude Bernard Teaching Hospital and by the Institutional Review Board (Patient’s Protection Committee, CPP) of the Ile-de-France region where both hospitals are located.

**Table 1:** Characteristics of the 82 participating healthcare workers and 54 study patients.

PATIENTS (n = 54)		Total duration of airborne precautions (days)
**Sex**, n (%)		
Male	38 (70)	
Female	16 (30)	
**Age**, years, median (IQR)	43.0 (32.7–53.1)	
**TB status**, n (%):		
AFB-positive sputum smear	6 (11)	149
Documented TB without AFB-positive sputum smear	3 (6)	15
Airborne precautions and empirical TB treatment	5 (9)	50
Airborne precautions for suspected but unconfirmed TB	40 (74)	190
**HEATHCARE WORKERS (n = 82)**		
**Sex**, n (%):		
Male	13 (16)	
Female	69 (84)	
**Age**, years, median (IQR)	32.0 (26.5–40.8)	
**Job category**, n (%):		
Nurse	29 (35)	
Nursing assistant	21 (26)	
Medical student	12 (15)	
Resident	9 (11)	
Senior physician	6 (7)	
Other	5 (6)	

Abbreviations: TB, tuberculosis; AFB, acid-fast bacilli; IQR, interquartile range.

### Patients

Only interactions with patients included in the study were analyzed. Patients were included if they had suspected or confirmed TB as defined by any of the following: AFB-positive sputum-smear examination, AFB-negative sputum-smear examination but bacteriologically-confirmed TB, or airborne isolation precautions pending results of an AFB sputum-smear examination. Patients were included only for as long as airborne isolation lasted, excluding the first and last day, because the times of initiation and discontinuation of airborne precautions were not collected. For each patient, the following information was collected: unit and room, dates of admission and discharge, dates of starting and stopping airborne isolation precautions, and results of the AFB sputum-smear examination and bacteriological culture for *Mycobacterium tuberculosis*.

**Figure 2:**
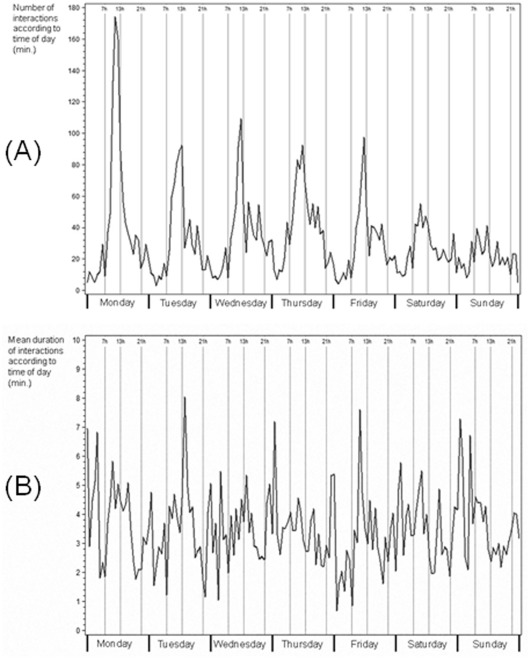
Distribution of (A) number per hour and (B) mean duration per hour of 5490 interactions between 82 healthcare workers and 54 patients placed under airborne precautions over one week during the study period. Footnote: The graphs show cumulative data from 5490 interactions during the 3-month study period in both study wards.

### Deployment of the Radio Frequency Identification Device

We deployed a RFID in both clinical units (i.e., 50 patient rooms), first in the infectious diseases ward for 3 months then in the pulmonology ward for another 3-month period. RFID nodes included fixed devices in patient rooms and mobile devices carried by HCWs. Because of energy constraints and for simplicity, we used an asymmetric network. Each room was equipped with a fixed sensor that continuously listened to radio signals. The sensor was secured to the television arm at a height of 2.5 meters and was plugged into a power outlet. Each HCW carried an autonomous mobile sensor in a pocket of their uniform. Each mobile sensor weighed about 30 grams and was about the size of a pager. Mobile sensors were programmed to emit a radio packet containing their unique identification number, at intervals of 5 seconds. Radio signals emitted by mobile sensors were recorded by nearby fixed sensors, according to a four-level intensity scale (from lowest, 1; to highest, 4). Signal intensity reflected the distance between the mobile and fixed sensors and the presence of an obstacle between the two sensors. The asymmetric deployment of the RFID simplified communication between nodes and guaranteed privacy for the HCWs. The method for sensor deployment has been described in detail elsewhere [4].

### Signal Reconstruction

All interactions between mobile HCW sensors and fixed sensors in study patient rooms were collected during the study period. Signal reconstruction was performed to discard false-positive signals and to restore false-negative signals. False-positive signals could occur if the HCW stayed in front of the open door but outside the patient’s room and false-negative signals if the body of the HCW was between the mobile sensor and the fixed sensor. Consequently, of the mobile-sensor signals emitted every 5 seconds, only those received at intensity levels of 3 or 4 were used to reconstruct actual interactions [4]. The results were validated by comparison with data from direct observation during a validation phase. In addition, long interaction durations recorded by the RFID were routinely compared to the patient’s chart and nurse log.

**Table 2:** Interactions between 82 healthcare workers and 54 patients placed under airborne precautions.

		Morning (7 a.m.–1 p.m.)			Afternoon (1 p.m.–9 p.m.)		Night (9 p.m.–7 a.m.)			*P* value	
Total number		2480			1905		1105				
Total number/hour		413			238		110				
Duration of each interaction (min.), median (IQR)		2.3 (0.8–5.0)			1.8 (0.7–3.7)		2.0 (0.7–4.3)			<10^−4^	
	**Nurse** **n = 29**		**Nursing assistant** **n = 21**	**Resident** **n = 9**		**Medical student** **n = 12**		**Senior physician** **n = 6**	**Others HCWs** **n = 5**		***P*** ** value**
**Interactions** (n):											
Total number	1899		1393	1125		804		209	60		
Total number/HCW	65		66	125		67		35	12		
**Duration of each interaction** (min.), median (IQR)	1.7 (0.7–3.8)		1.9 (0.7–4.1)	2.5 (0.9–5.5)		2.7 (1.0–6.0)		1.9 (0.8–4.1)	1.7 (0.5–2.8)		<10^−4^

Abbreviations: IQR, interquartile range; HCW, Healthcare worker.

### Direct Observation and Healthcare-worker Interviews

During the period of RFID deployment, direct observation of patient-HCW interactions was performed and HCWs were interviewed about their recollections of interactions with study patients.

Direct observation was performed during day shifts. Each entry of any HCW into a study patient room was recorded over periods of 20 to 30 minutes. The following data were collected: name of the HCW, room number, identification of the fixed and mobile sensors, and time spent by the HCW in the room.

Perceptions of interactions by HCWs were collected by interviewing HCWs 1 to 7 days after contact with a study patient. HCWs were asked to report the perceived frequency of room entries during a specific day and the total time spent in the room during their shift.

### Statistical Analysis

Time spent by HCWs in patient rooms was described as median (interquartile range, minimum and maximum value). These durations were compared across job categories and shifts using the non-parametric Kruskal-Wallis test. To compare durations recorded by the RFID to durations determined from paired direct observations and HCW interviews, we used the non-parametric paired Wilcoxon test. All tests were two-sided at the 0.05 significance level. All analyses were performed with SAS version 9.2 (SAS Institute, Cary, NC, USA).

To assess agreement between RFID-recorded interactions and either direct observation or interview data, we constructed Bland-Altman plots [5].

**Figure 3:**
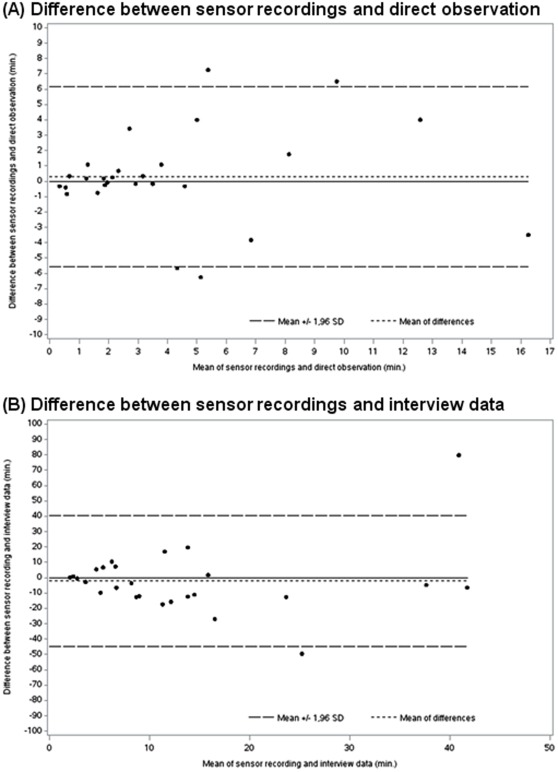
Bland-Altman plots of paired interactions. (A) Difference between sensor recordings and direct observation; (B) Difference between sensor recordings and interview data.

## Results

### Healthcare Workers and Patients

Of the 70 and 54 HCWs working in the two study units, respectively, 65 (93%) and 46 (85%), respectively, agreed to participate. Of these 111 HCWs, 82 had at least one interaction with at least 1 patient placed under airborne precautions (Figure 1), including 29 (35%) nurses, 21 (26%) nursing assistants, 12 (15%) medical students, 9 (11%) residents, 6 (7%) fellows or senior physicians, and 5 (6%) other HCWs.

Of the 64 patients placed under airborne precautions, 10 had no contact with a participating HCW during the study period. Of the 54 remaining patients, 6 (11%) had AFB-positive sputum-smear tests, 3 (6%) had documented TB without AFB-positive sputum-smear tests, 5 (9%) were given empirical TB treatment and 40 (74%) had suspected but unconfirmed TB (Table 1).

In total, 404 isolation days were assessed, with 5490 interactions between the 82 HCWs who had at least one interaction with at least one of the 54 study patients during the 3-month study period in each unit.

### Interactions

We first plotted the number per hour and duration per hour of each patient-HCW interaction according to time of day, after signal reconstruction over 1 week during the study period. Figure 2A reports the global distribution by week days of interactions according to time of day. Most interactions occurred during the first weekdays, with a peak between 11 a.m. and 1 p.m. Mean interaction duration according to time of day was evenly distributed over the week (Figure 2B).

Overall, data were obtained for a median of 65.0 (IQR, 36.0–86.0) shifts per HCW over the 3-month period. The median total number of interactions with patients was 20.0 (IQR, 7.0–123.0) per HCW and the median total duration of interactions was 57.3 (IQR, 13.7–379.6) minutes per HCW. The median number and duration of interactions per shift were 0.4 (IQR, 0.1–1.5) and 1.1 (IQR, 0.4–5.9) minutes, respectively.

The median duration per HCW-patient interaction was 2.1 (IQR, 0.8–4.4) minutes overall, 2.3 (IQR, 0.8–5.0) minutes during the morning shift (7 a.m.–1 p.m.), 1.8 (IQR, 0.7–3.7) minutes during the afternoon shift (1 p.m.–9 p.m.), and 2.0 (IQR, 0.7–4.3) minutes during the night shift (9 p.m.–7 a.m.) (*P*<10^−4^). The number of interactions per hour was higher in the morning (n = 413) than in the afternoon (n = 238) or at night (n = 110) (Table 2).

The median total time spent by each HCW interacting with all TB patients was 7.6 minutes (IQR, 2.4–22.5 minutes; range, 0.2 minutes-5.3 hours) and the median number of interactions/day/HCW was 3.0 (IQR, 1.0–6.0).

The total number of interactions was 1899 for nurses (n = 65 per nurse), 1393 for nursing assistants (n = 66 per nursing assistant), 1125 for residents and senior physicians (n = 125 per physician), and 804 for medical students (n = 67 per student). Median interaction duration varied significantly across job categories (*P*<10^−4^) (Table 2).

### Direct Observation and Healthcare-worker Interviews

Of the 79 direct observation periods, 67 were performed during the 404 days of assessed airborne precautions, and 28 could be evaluated. The 39 missing pairs were related to nonparticipation of observed HCWs in the study, absence of carrying the sensor by the HCW, failure of signal reconstruction if the HCW-stay in the room was brief or interrupted with several entries and exits, or technical sensor failure. Median interaction duration by direct observation was 2.0 (IQR, 1.5–5.6) minutes and was not significantly different from the corresponding value collected by the RFID (2.5 [IQR, 1.6–4.7] minutes; *P* = 0.6).

Of 91 HCW interviews about the number and duration of interactions between the interviewed HCW and a specific TB patient on a specific day, 26 could be evaluated. The remaining 65 interviews were not assessable, for the same reasons as listed above for direct observation. Median daily interaction duration as perceived by the HCWs was 10.0 (IQR, 3.0–20.0) minutes, which was not significantly different from the median duration collected by the RFID (6.8 [IQR, 2.6–16.7] minutes; *P* = 0.2).

The Bland-Altman plots of the difference between RFID-recorded interactions and paired direct observations or interviews are shown in Figure 3A and 3B, respectively. Agreement was good, although variability was greater for long than for short interactions.

## Discussion

We deployed a large RFID in two clinical units to measure interactions between HCWs and patients with TB. Calibration and signal reconstruction allowed us to obtain data on interaction duration, as confirmed by the comparison with direct observation data during the validation phase. Although daily median interaction duration per HCW and per TB patient was short, i.e., 7.6 minutes, 25% of interactions were longer than 22.5 minutes and some interactions lasted several hours. Physicians and medical students were exposed as frequently but for longer times, compared to nurses and nursing assistants. Finally, perceptions collected by HCW interviews accurately reflected exposure to TB patients.

Most data on interactions between HCWs and patients are obtained via direct observation by trained observers. Direct observation data may be biased, however, chiefly via the Hawthorne effect. The Hawthorne effect is a change in the behavior of people who know they are being observed. Although not systematically evaluated, several studies in the field of hand hygiene suggested overestimation of compliance related to the Hawthorne effect [6], [7]. Other biases are observer bias, with observers from the audited unit tending to report higher compliance; and selection bias, with direct observation being performed in units or during periods believed to be characterized by better compliance [8]. In addition, direct observation is time-consuming. However, the World Health Organization considered direct observation to be the reference standard for assessing hand-hygiene compliance rates.

Social network specialists and infection control specialists recently developed automatic systems for collecting interactions between HCWs and patients. These systems have proved helpful for assessing contact patterns and studying the spread of respiratory viruses, with the goal of setting up prevention strategies [9], [10], [11]. Also, compliance with hand hygiene at room entry and exit can be measured using electronic devices [12]. Electronic methods allow continuous and automatic collection of person-to-person interactions in hospitals [10]. For our study, we selected tuberculosis because of its epidemiological simplicity, with a single reservoir and exclusive airborne transmission. Moreover, much remains to be learned about the transmission of *M. tuberculosis* to HCWs.

We had some concern that the HCWs might perceive the sensors as intrusive. However, the study was well accepted, with a high participation rate. We were therefore able to describe interactions between HCWs and patients in two large clinical units. However, we encountered unexpected difficulties in calibrating and reconstructing interactions. Obstacles to the collection of accurate data included technical issues and problems related to real-life conditions (e.g., the body of the HCW shielding the fixed sensor from the emissions by the mobile sensor). These technical aspects have been reported previously [4]. Our experience shows that investigators who intend to implement an electronic system for describing social interactions should consider potential obstacles and plan a detailed calibration phase.

Interactions during the morning shift (7 a.m.–1 p.m.) were about twice as numerous as those during the afternoon shift (1 p.m.–9 p.m.) and four times more numerous than during the night shift (9 p.m.–7 a.m.). In addition, interaction duration was significantly longer during the morning shift. This distribution is in accordance with the organization of care in the wards.

The median duration of each interaction was only 2.1 minutes. This is not surprising, as TB patients are usually self-sufficient and do not require major nursing procedures. However, daily care duration was as long as several hours in some TB patients, suggesting a risk of TB transmission to HCWs in the absence of airborne isolation precautions. However, most HCWs had short interaction durations. These data should prove helpful for investigations by occupational physicians working on risks to HCWs. The interaction duration cutoff above which a contact investigation should be considered after exposure to an infectious TB patient in the absence of airborne isolation precautions varies widely across recommendations, from 1 hour in France to 8 hours for aircraft passengers in close contact with the case [3]. Our results in two hospital wards show that prolonged contact is rare.

Our finding of longer interactions between TB patients and medical students and residents, compared to HCWs in other job categories, indicates a need for including students and residents into contact investigations. Medical students may not be under monitoring by the hospital occupational physicians and may therefore fail to receive active surveillance after exposure.

The good correlation between direct observation data and RFID data confirmed the accuracy of the recorded interactions. HCW perceptions of daily interaction durations were accurate during interviews conducted after 1 to 7 days. However, some correlations between RFID data and data from either direct observation or interviews were weaker, especially for long interactions, suggesting that signal reconstruction may not always be accurate (presence of false-positive and false-negative signals despite signal reconstruction). Technological improvements will be available soon, such as ultra-wideband radio signals, and will improve indoor sensor network localization.

To our knowledge, only one study evaluated interactions between HCWs and patients in a pediatric clinical ward. The authors obtained a high participation rate from HCWs, as in our study. Although not validated by direct observations, the results were essentially similar to our data, with a short median duration of each interaction between participants and large variations of the number and duration of contacts between participants and within job categories [10].

In conclusion, this preliminary study demonstrates that interactions between HCWs and patients can be recorded accurately and continuously using a RFID. Electronic sensors can be used as an alternative or as a complement to time-consuming short-term direct observation. RFIDs hold promise for better describing exposure and risk, provided correct calibration is performed before data acquisition and appropriate signal reconstruction methods are used. RFIDs could be used for assessing the risk of transmissible infectious diseases.
